# Dietary Advanced Glycation End Products and Risk of Overall and Cause-Specific Mortality: Results from the Golestan Cohort Study

**DOI:** 10.3390/ijerph20053788

**Published:** 2023-02-21

**Authors:** Elham Hosseini, Zeinab Mokhtari, Hossein Poustchi, Masoud Khoshnia, Sanford M. Dawsey, Paolo Boffetta, Christian C. Abnet, Farin Kamangar, Arash Etemadi, Akram Pourshams, Maryam Sharafkhah, Paul Brennan, Reza Malekzadeh, Azita Hekmatdoost

**Affiliations:** 1 Nutrition and Food Security Research Center, Department of Clinical Nutrition, School of Nutrition and Food Science, Isfahan University of Medical Sciences, Isfahan 81746-73461, Iran; 2 Liver and Pancreaticobiliary Disease Research Center, Digestive Diseases Research Institute, Shariati Hospital, Tehran University of Medical Sciences, Tehran 14117-13135, Iran; 3 Digestive Oncology Research Center, Digestive Diseases Research Institute, Shariati Hospital, Tehran University of Medical Sciences, Tehran 14117-13135, Iran; 4 Golestan Research Center of Gasteroenterology and Hepatology (GRCGH), Golestan University of Medical Sciences, Gorgan 49178-67439, Iran; 5 Metabolic Epidemiology Branch, Division of Cancer Epidemiology and Genetics, National Cancer Institute, Bethesda, MD 20892, USA; 6 Icahn School of Medicine at Mount Sinai, New York, NY 10029, USA; 7 Department of Biology, School of Computer, Mathematical, and Natural Sciences, Morgan State University, Baltimore, MD 21251, USA; 8 Genetic Epidemiology Group, International Agency for Research on Cancer (IARC/WHO), CEDEX 07, 69366 Lyon, France; 9 Department of Clinical Nutrition and Dietetics, Faculty of Nutrition and Food Technology, National Nutrition and Food Technology Research Institute, Shahid Beheshti University of Medical Sciences, Tehran 19816-19573, Iran

**Keywords:** cohort, advanced glycation end products, dietary intake, mortality, cancer

## Abstract

Controversy exists regarding the association of dietary advanced glycation end products (dAGEs) with the risk of disease outcomes and mortality. We aimed to examine, prospectively, the association between dAGEs intake and the risk of overall and cause-specific mortality in the Golestan Cohort Study. The cohort was conducted between 2004 and 2008 in Golestan Province (Iran) recruiting 50,045 participants aged 40–75 years. Assessment of dietary intake over the last year was performed at baseline using a 116-item food frequency questionnaire. The dAGEs values for each individual were calculated based on published databases of AGE values of various food items. The main outcome was overall mortality at the time of follow-up (13.5 years). Hazard ratios (HRs) and 95% confidence intervals (CIs) for overall and cause-specific mortality were estimated according to the dAGEs quintiles. During 656, 532 person-years of follow-up, 5406 deaths in men and 4722 deaths in women were reported. Participants at the highest quintile of dAGE had a lower risk of overall mortality (HR: 0.89, 95% CI: 0.84, 0.95), CVD mortality (HR: 0.89, 95% CI: 0.84, 0.95), and death from other causes (HR: 0.89, 95% CI: 0.84, 0.95) compared to those in the first quintile after adjusting for confounders. We found no association of dAGEs with risk of mortality from cancer (all), respiratory and infectious diseases, and injuries. Our findings do not confirm a positive association between dAGEs and the risk of mortality in Iranian adults. There is still no agreement among studies investigating dAGEs and their health-related aspects. So, further high-quality studies are required to clarify this association.

## 1. Introduction

Advanced glycation end products (AGEs) are a diverse group of compounds formed as the end products of spontaneous glycation of amino groups of amino acids through the non-enzymatic Millard reaction [1]. During the heat processing of foods, the Millard reaction occurs when the carbonyl group of reducing sugars interacts with the amino acid of peptides or proteins, resulting in the reversible formation of Schiff base compounds that can promptly undergo molecular rearrangements to so-called Amadori products [2]. The Amadori products are pertinent precursors for AGEs as they can rearrange into AGEs [2]. The Schiff base compounds or the Amadori product precursors can also be degraded into reactive dicarbonyls such as methylglyoxal, glyoxal, and 3-deoxyglucosone. These reactive dicarbonyls can react with a free or bound amino acid and form AGEs [2]. If excessive amounts of AGEs reach tissue and circulation, they become pathogenic [1]. This can occur by consuming a diet containing animal-source foods and cooking processes, in particular roasting, grilling, boiling, and frying, resulting in a further formation of AGEs in foods [3]. Diets with high AGEs content have been associated with cardiovascular diseases (CVD) and metabolic dysfunction [4,5,6].

Similar non-enzymatic reactions, as described above, occur during the normal glycation process of the cell in human tissues to form AGEs, but at lower rates due to the lower physiological temperature [7]. Additional endogenous AGE formation pathways include glycolysis and the polyol pathway. In glycolysis, glyceraldehyde 3-phosphate produced through the general metabolism of glucose or fructose can spontaneously decompose to the reactive dicarbonyl compound methylglyoxal, resulting in AGEs formation [7]. The polyol pathway is active under hyperglycemic conditions and requires glucose conversion to sorbitol and sorbitol conversion to fructose, promoting the accumulation of dicarbonyl compounds and AGEs [7]. Moreover, lipid peroxidation of polyunsaturated fatty acids in cell membranes can also lead to increased dicarbonyl production and subsequent AGE formation [7]. The role of endogenous AGEs in various diseases and conditions, including diabetes and its microvascular complications, neurodegenerative disorders, some cancers, bone diseases, and oxidative stress conditions and chronic inflammation, has been explored [1,8].

Two major mechanisms are attributed to the pathologic effect of AGEs: Firstly, they may conjoin proteins and directly change their structure and consequently their features and function. Secondly, AGEs bind to a specific receptor assigned as the receptor for AGEs (RAGE), which is a multi-ligand receptor and therefore binding of AGE ligands to the receptor can result in stimulation of the proinflammatory transcription factor nuclear factor-kappaB, inducing oxidative stress and inflammatory conditions [9].

The possible effect of dietary AGEs (dAGEs) on human health was previously ignored because it was believed that dietary AGEs are only slightly absorbed [3]. However, experimental studies with diets rich in AGEs have indicated a positive correlation between dAGEs and the body’s AGE pool [10]. A higher intake of dAGEs increased the chance of general and abdominal obesity as the main risk factors for several chronic diseases [11,12]. In a prospective cohort study, higher dAGEs intake increased the risk of breast cancer in postmenopausal women [13]. Consumption of dAGEs promoted the growth of breast and prostate tumor models by forming a tumor-promoting stromal microenvironment [14]. Although some studies have investigated the association of dAGEs and chronic disease mortality in healthy populations, as well as adults with co-morbidities, little is known about the ability of dAGEs for predicting all and cause-specific mortality in a general adult population. To our knowledge, no study has investigated the association of dAGEs intake with the risk of overall mortality in Iran. Therefore, we aimed to examine, prospectively, the association between dAGEs and risk of overall mortality in an Iranian population. The association of dAGEs with the risk of CVD and cancer mortality was also investigated.

## 2. Methods

### 2.1. Background

**Figure 1:**
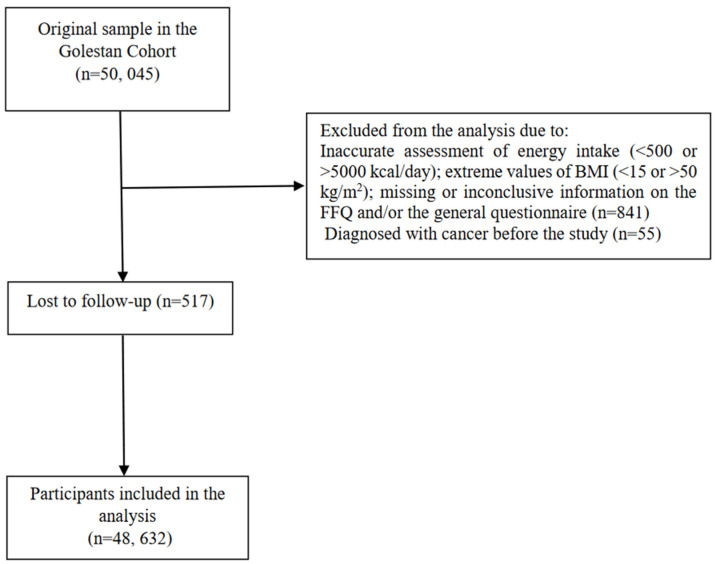
The GCS flow diagram showing the numbers of people who were included in the analyses. The GCS, the Golestan Cohort Study; BMI, body mass index; FFQ, food frequency questionnaire.

We examined data from the Golestan Cohort Study (GCS), a population-based cohort of the general population in the Golestan Province, in Northeast Iran. The design of the GCS has been previously described elsewhere [15]. In summary, the cohort aimed to investigate the incidence of oesophageal squamous cell carcinoma. The study was conducted between 2004 and 2008 in Golestan Province, recruiting 50,045 participants aged 40–75 years, from Gonbad city and 326 rural areas (20% and 80% from urban and rural areas, respectively). Each participant was provided with an informed consent form before enrollment. Participants were excluded if they had an inaccurate assessment of energy intake, were diagnosed with cancer before the study, missing or inconclusive information on the food frequency questionnaire (FFQ) and/or the general questionnaire (containing information on socio-demographic and socio-economic status, history of diabetes and hypertension, smoking, alcohol drinking, opium use, and anthropometrics), and extreme values of body mass index (BMI). In total, 48,632 individuals were included in our analyses (27,975 women and 20,657 men) (Figure 1). The Institutional Review Boards of the Digestive Disease Research Center (DDRC) of Tehran University of Medical Sciences, the US National Cancer Institute (NCI), and the World Health Organization International Agency for Research on Cancer (IARC) approved the study.

### 2.2. Dietary Assessment

The FFQ from the GCS was used to assess the usual frequency and portion size of dietary intake of 116 food items over the past 12 months. The questionnaire was found reliable and valid [16]. Data on usual portion size, consumption frequency, and servings consumed each time was obtained for each food item at recruitment. Consumption frequency of each food item was questioned according to a daily, weekly, or monthly basis and converted into daily intakes; portion sizes were then converted into grams using household gauges [17,18]. Nutritionist V software and the Iranian Food Composition Table [19] were used to assess daily dietary intake. To estimate the dAGEs intake of different foods including fruits, vegetables, dairies, cereals, meats (white and red meat) and processed meats (sausage, hamburger, salted fish, and smoked fish), and fats, published databases of AGE values of various food items were used to calculate a weighted mean value of dAGE in each FFQ line item [3,20]. We used published databases of the AGE content of commonly consumed foods because there is no information available on AGE values in the Iranian Food Composition Table [19]. In these databases, the AGEs content of 549 food items was measured using a validated immunoassay method [3,20] and data were available for N^ε^-carboxymethyllysine (CML) as the most-studied AGE in literature. We defined the CML values in kilo-Unit (kU) per 100-g solid food or 100 milliliters of liquid for 84 food items. For each food item, the individual AGE value was calculated by multiplying the assigned CML value by the frequency and portion size (gram value of the respective food item) reported by the individual. The total dAGE value for each participant was then calculated as the sum of the individual AGE values of various food items included in the FFQ. Food items with no similar food available in the databases were considered missing (32 food items) [11]. Because AGEs values were not available for all kinds of fruits, vegetables, and legumes, the mean values of comparable fruits, vegetables, and legumes were considered [11,12].

### 2.3. Measurement of Potential Confounding Variables

All participants were interviewed by instructed clinicians and/or non-clinicians, and data on lifestyle and demographics were obtained using a pre-defined questionnaire. Anthropometrics including weight, height, BMI, and waist-to-hip ratio (WHR) were taken based on the World Health Organization guidelines [15,21]. Physical activity was expressed in the metabolic equivalent of task per minute per week and grouped into tertiles [22]. Wealth score was a proxy of socioeconomic status and was estimated for each participant based on house ownership, structure, size and appliances, family size, etc. [23]. Data on wealth scores were then categorized into quartiles. Other potential confounders included age, gender, cigarette smoking, opium use, alcohol drinking, and history of diabetes and hypertension.

### 2.4. Follow-Up and Cause of Death Ascertainment

Follow-up strategies of this cohort study have been detailed elsewhere [15]. In summary, follow-ups were performed every 12 months. The vital status of the participants was obtained through phone calls or home visits by the study group. The overall success rate at the time of follow-up (13.5 years) was 98.9% (517/50,045 lost to follow-up). The main outcome was all-cause mortality. Any death report was affirmed by a clinician visit and a complete validated verbal autopsy questionnaire [24]. Moreover, two external internists separately investigated all information regarding the verbal autopsy and medical records and recognized the cause of death. In case of any disagreement between the two specialists, a third, more proficient internist considered all data and made the ultimate decision [15]. For the analyses, major causes of death among the participants were assessed as the secondary outcomes. Analyses were performed only on subjects with affirmed death.

### 2.5. Statistical Analysis

Total dAGE values were categorized into quintiles and the characteristics of participants were compared across the quintiles of dAGE. Analysis of variance (ANOVA) statistical analysis and the χ^2^ test for continuous and categorical variables were used to compare the characteristics of participants across the quintiles of dAGE. Cox proportional hazard models with follow-up duration as the timescale and dAGE quintiles as the exposure, with the lowest category as the reference, were used to assess the associations between dAGE and risk of overall and cause-specific mortality. In the Cox models, age and multivariate-adjusted hazard ratios (HRs) and 95% confidence intervals (CIs) were provided for each outcome. In the multivariate models, the HRs were adjusted for confounding variables, including age, gender, energy intake, physical activity, pack-years of cigarette smoking, BMI, alcohol drinking, opium use, and history of diabetes and hypertension. The length of follow-up for each participant was considered from the recruitment date to the study until the date of death, lost to follow-up, or the reference follow-up date (30 July 2018), whichever arose first. All the statistical analyses were carried out in SPSS (version 18; SPSS Inc., Chicago, IL, USA) and *p* < 0.05 was regarded as significant.

## 3. Results

In total, 48,632 participants were included in our analysis, of which 57.5% were women and 79.7% were inhabitants of rural areas. The average age (standard deviation (SD)) of participants at baseline was 52 (8.9) years. During 13.5 years (3.4) of follow-up, 10,128 deaths were documented (46.6% women). The main causes of death were cardiovascular diseases (3762), gastrointestinal cancer (966), other cancers (815), respiratory diseases (648), infectious diseases (418), injuries (402), and other causes (1527).

As presented in Table 1, participants at the highest quintile of dAGE values were younger, had higher BMI and WHR, and were more likely to smoke compared with those at the lowest quintile. There were also more alcohol drinkers, and fewer reports of a history of diabetes and hypertension among the participants at the highest quintile of dAGE. Moreover, compared with those at the lowest quintile, participants at the highest quintile of dAGE had higher wealth scores and more energy intake. Calculated total dAGE values for all food items in FFQs ranged from 67.6 to 21,995.9, and the mean dAGE value (SD) of all participants was 7066.7 (2916.8). Participants with higher dAGEs tended to consume more fruits, vegetables, dairy, cereals, meats, and fats (Table 1).

Table 2 presents HRs for all-cause mortality, according to the dAGE quintiles. Participants at the highest quintile of dAGE had a lower risk (age-adjusted) of all-cause mortality (HR: 0.86, 95% CI: 0.81, 0.92) compared to those in the first quintile. Further adjustment for other confounding variables including energy intake, physical activity, smoking, BMI, alcohol drinking, opium usage, and history of diabetes and hypertension did not change the results (Table 2).

Table 3 presents HRs for cause-specific mortality, according to the dAGE quintiles. Participants at the highest quintile of dAGE had a lower risk of CVD mortality (HR: 0.88, 95% CI: 0.79, 0.98) compared to those in the first quintile, and the decreased risk was more evident in women. Adjusted HRs indicated no association of dietary dAGE intake with risk of mortality from all cancer (HR: 0.89, 95% CI: 0.76, 1.03), gastrointestinal (HR: 0.89, 95% CI: 0.72, 1.09), and other cancers (HR: 0.89, 95% CI: 0.71, 1.11). These findings did not differ in sex-specific analyses. Participants at the highest quintile of dAGE had a lower risk of death from other causes (than all cancers, respiratory and infectious diseases, and injuries) (HR: 0.79, 95% CI: 0.67, 0.92) compared to those in the first quintile, and the decreased risk was more evident in men. No association was observed between dAGE quintiles and death from infectious and respiratory diseases and injuries (Table 3).

## 4. Discussion

Examining longitudinal data from the GCS, we did not find dAGEs to be associated with an increased risk of overall and cause-specific mortality. We observed that a higher intake of dAGEs was associated with a reduced risk of overall mortality, CVD mortality, and death from other causes. A gender-specific analysis showed that the highest versus lowest quintiles of dAGEs in men were in association with a 12% and 24% reduced risk of overall mortality and death from other causes, respectively. Compared to the lowest quintile, women at the highest quintile of dAGEs had 9% and 19% lower risk of overall and CVD mortality, respectively.

The findings of the present study are in agreement with the recent study of Nagata et al. [25], who showed that a higher intake of CML, a major AGEs product, was inversely associated with the risk of total mortality in Japanese adults. Furthermore, no association was found between dietary intake of AGEs and total and colorectal cancer mortality among colorectal cancer patients in the EPIC (European Prospective Investigation into Cancer and Nutrition) study [26]. Similar findings have been reported when examining the association of serum AGEs and all-cause and CVD mortality [27]. Dissimilarly, adolescents with the highest dAGE intake were more likely to have metabolic syndrome when compared to the lowest quartile of dAGE intake [28]. In a large prospective cohort during the period of 12.8-year follow-up, higher dAGE intake was associated with increased risk of breast cancer in postmenopausal women [13]. Moreover, higher dAGEs have been related to the increased risk of all-cause, and CVD and breast cancer mortality in postmenopausal women diagnosed with invasive breast cancer [29]. In another study, during the follow-up of 10.5 years, men but not women in the fifth quintile of dAGE intake had higher risk of pancreatic cancer [30]. In the course of 13-year follow-up, no significant association was revealed between higher CML intake and the total cancer risk in male and female participants [31]. Yet, CML intake at the highest quartile was associated with the increased risk of liver cancer, while it was associated with the decreased risk of male stomach cancer [31]. One explanation for the contradictory results is the inconsistency of the AGE content of foods or diets used in different studies due to different cooking processes. Besides, population characteristics per se might affect the association as well. The majority of studies were performed on subjects with preexisting medical conditions which could affect the results when compared to healthy adults or the general population.

Controversy exists regarding the toxicity of AGEs in the body. In observational studies, higher dAGEs have been associated with intermediate outcomes such as oxidative stress and inflammation in type 2 DM patients [4]. In subjects with cardio-metabolic diseases such as overweight, obesity, or prediabetes, an AGE-restricted diet reduced some inflammatory markers and improved insulin sensitivity [5]. However, a meta-analysis of clinical trials did not support the effect of AGE-restricted diets on the inflammatory profile of healthy individuals and those with diabetes or renal failure [32]. On the other hand, a positive association of dAGEs with chronic disease outcomes such as breast cancer [13], obesity [12], and chronic kidney disease [33] has been shown. The toxicity effect might originate from the studies in which the dietary content of AGEs is a significant contributor to the excess serum AGEs levels [34]. This toxicity, however, has been debated in the literature [35].

Studies examining the association of dAGEs and total and/or cause-specific mortality are rare, and thus there is no conclusive evidence suggesting dietary AGEs to be detrimental to human health [36]. A major part of the AGE content of foods absorbed is rapidly excreted by kidneys, resulting in insignificant plasma levels of these metabolites [37]. Due to the very rapid excretion of CML from the body, the probability of any effect on body proteins has been considered to be low, and therefore should have only limited consequences in some organs such as the liver and kidneys [37]. Therefore, the effect of dAGEs on human health still needs further elucidation.

Our results showed that higher dAGE values were less protective in men, regarding the association of dAGEs and risk of CVD mortality, compared to female participants. This could be explained by some additional CVD risk factors such as age above 50, smoking, alcohol drinking, and opium use being more frequent in men. On the contrary, compared to the lowest quintile of dAGE, men with higher dAGE values had a lower risk of total mortality and death from respiratory diseases, probably due to the lower BMI and WHR and more physical activity compared to women.

Our study has several strengths including the longitudinal design, the large sample size representing the general population, and a high rate of follow-up. Additionally, we performed our analyses by adjusting for the most relevant confounders. There are some limitations as well. The first was dAGEs values considered for each food item from the beginning. Since there is no AGE value available for any food item in Iranian food composition tables, we used the most commonly studied AGE databases based on diets common in a Northeastern Metropolitan US area [3,20], which might not represent the Iranian foods estimated in this study. Moreover, even for similar food items, the AGE content of food measured in literature might differ from the AGE content of food items in the FFQ used in the present study due to different cooking processes and could, therefore, affect the results. Secondly, we considered the same AGE values for some similar food items such as fruits, legumes, and vegetables for which no respective AGE values were available in the literature. Additionally, some characteristics of subjects might have changed since baseline measurement and would therefore affect the analyses.

In conclusion, our findings indicated an inverse association between dAGEs intake and the risk of overall and cause-specific mortality. Although it has been shown that dietary AGEs are associated with an increased risk of diseases, our findings did not confirm a positive association between dAGEs and mortality in Iranian adults. There is still no agreement among studies investigating dAGEs and their health-related aspects. Evidence has either debated against the adverse effects of dAGEs or revealed a protective effect of an AGE-restricted diet on different health conditions for some specific dAGEs due to antioxidant activity. Yet, studies on healthy subjects are limited and current evidence is indecisive. So, further high-quality studies are required to clarify the impact of dietary AGEs on disease and mortality risk.

## Figures and Tables

**Table 1:** Baseline characteristics of participants according to the quintiles of dAGE ^a^.

	dAGE Value				
	Q1	Q2	Q3	Q4	Q5
	(n = 9674)	(n = 9758)	(n = 9740)	(n = 9738)	(n = 9722)
Men %	3910 (40.4)	4121 (42.2)	4123 (42.3)	4244 (43.6)	4259 (43.8)
Age (year) ^b^	53.18 ± 9.35	51.76 ± 8.74	51.58 ± 8.64	51.53 ± 8.71	52.13 ± 8.92
BMI (kg/m^2^) ^b^	26.23 ± 5.54	26.56 ± 5.41	26.77 ± 5.36	26.88 ± 5.28	26.99 ± 5.32
Waist-to-hip ratio ^b^	0.95 ± 0.08	0.95 ± 0.08	0.96 ± 0.08	0.96 ± 0.08	0.96 ± 0.08
Smoker ever %	1624 (16.8)	1680 (17.2)	1635 (16.8)	1710 (17.6)	1757 (18.1)
Pack-years of cigarette smoking					
%					
0	8050 (83.2)	8078 (82.8)	8105 (83.2)	8028 (82.4)	7965 (81.9)
0.1–5	535 (5.5)	525 (5.4)	523 (5.4)	546 (5.6)	571 (5.9)
5.1–10	210 (2.2)	275 (2.8)	236 (2.4)	275 (2.8)	250 (2.6)
10.1–20	342 (3.5)	354 (3.6)	352 (3.6)	354 (3.6)	367 (3.8)
20 <	537 (5.6)	526 (5.4)	524 (5.4)	535 (5.5)	569 (5.9)
Alcohol ever used % ^b^	288 (3.0)	300 (3.1)	336 (3.4)	371 (3.8)	386 (4.0)
Opium ever used % ^b^	1742 (18.0)	1641 (16.8)	1533 (15.7)	1584 (16.3)	1719 (17.7)
History of diabetes % ^b^	819 (8.5)	615 (6.3)	565 (5.8)	623 (6.4)	740 (7.7)
History of hypertension % ^b^	2168 (22.4)	1878 (19.2)	1849 (19.0)	1787 (18.4)	1921 (19.8)
Wealth score % ^b^					
• 1st quartile	3512 (36.3)	2938 (30.1)	2420 (24.8)	2292 (23.5)	2122 (21.8)
• 2nd quartile	2522 (26.1)	2318 (23.8)	2363 (24.3)	2183 (22.4)	1974 (20.3)
• 3rd quartile	1812 (18.7)	2328 (23.9)	2438 (25.0)	2608 (26.8)	2647 (27.2)
• 4th quartile	1828 (18.9)	2174 (22.3)	2519 (25.9)	2655 (27.3)	2979 (30.6)
Physical activity %					
• 1st tertile	3463 (35.9)	3378 (34.7)	3463 (35.6)	3447 (35.5)	3403 (35.1)
• 2nd tertile	3058 (31.7)	3081 (31.6)	2996 (30.8)	3048 (31.4)	3160 (32.6)
• 3rd tertile	3127 (32.4)	3280 (33.7)	3259 (33.5)	3222 (33.2)	3137 (32.3)
Total energy intake (kcal) ^b^	1869.4 ± 608.1	2075.7 ± 576.3	2156.6 ± 544.2	2260.1 ± 529.8	2454.8 ± 550.8
Fruits (g/d) ^b^	70.1 (52.6)	89.5 (55.3)	99.8 (57.2)	106.1 (58.6)	114.0 (61.5)
Vegetables (servings/d) ^b^	81.5 (43.8)	93.9 (43.4)	100.1 (42.6)	106.9 (43.6)	118.6 (46.7)
Dairies (g/d) ^b^	95.9 (83.4)	113.4 (83.8)	125.8 (84.5)	130.4 (82.7)	137.3 (83.2)
Cereals (g/d) ^b^	421.9 (154.2)	457.8 (151.4)	463.2 (150.3)	473.7 (148.7)	473.2 (150.2)
Meats and processed meats (g/d) ^b^	22.9 (15.5)	40.3 (19.3)	56.1 (21.5)	76.9 (24.9)	126.0 (44.4)
Fats (g/d) ^b^	16.1 (8.8)	24.6 (9.9)	27.9 (10.5)	30.9 (11.2)	34.8 (13.3)

dAGE, dietary advanced glycated end product; Q, quintile; BMI, body mass index. a Values are means ± SD for continuous variables and percentages for categorical variables. b These variables were statistically different across the dAGE quintiles (*p* < 0.05), ANOVA for quantitative variables and chi-square test for qualitative variables.

**Table 2:** Hazard ratios for all-cause mortality, according to the dAGE quintiles ^a^.

	dAGE Value					
						*p*-Value
	Q1	Q2	Q3	Q4	Q5	For Trend
Women						
No. of person-years	77,825	77,498	77,894	75,967	74,624	
No. of deaths	1164	972	869	810	907	
Model 1 ^b^	1.00	0.97 (0.89, 1.06)	0.87 (0.80, 0.95)	0.82 (0.75, 0.90)	0.85 (0.78, 0.93)	<0.001
Model 2 ^c^	1.00	0.99 (0.91, 1.08)	0.90 (0.82, 0.98)	0.86 (0.78, 0.94)	0.92 (0.84, 1.01)	0.005
Model 3 ^d^	1.00	1.01 (0.93, 1.10)	0.92 (0.84, 1.00)	0.88 (0.80, 0.97)	0.91 (0.83, 0.99)	0.011
Men						
No. of person-years	49,450	54,247	54,618	56,024	55,557	
No. of deaths	1260	1103	986	1016	1041	
Model 1 ^b^	1.00	0.91 (0.84, 0.98)	0.83 (0.76, 0.90)	0.83 (0.77, 0.90)	0.86 (0.79, 0.93)	<0.001
Model 2 ^c^	1.00	0.93 (0.85, 1.00)	0.85 (0.78, 0.92)	0.86 (0.79, 0.94)	0.89 (0.82, 0.97)	0.001
Model 3 ^d^	1.00	0.94 (0.86, 1.02)	0.86 (0.79, 0.93)	0.86 (0.79, 0.93)	0.88 (0.81, 0.96)	0.001
All						
Model 1 ^b^	1.00	0.95 (0.89, 1.00)	0.86 (0.81, 0.91)	0.84 (0.79, 0.89)	0.86 (0.81 0.92)	<0.001
Model 2 ^c^	1.00	0.96 (0.90, 1.02)	0.87 (0.82, 0.93)	0.86 (0.81, 0.92)	0.91 (0.85, 0.97)	<0.001
Model 3 ^d^	1.00	0.97 (0.92, 1.03)	0.88 (0.83, 0.94)	0.87 (0.82, 0.93)	0.89 (0.84, 0.95)	<0.001

DAGE, dietary advanced glycated end product; Q, quintile. ^a^ Cox proportional hazards regression models for estimating HRs and 95% CIs. ^b^ Model 1: adjusted for age. ^c^ Model 2: additionally, adjusted for gender (except when stratified by gender), energy intake, physical activity, smoking, BMI, and alcohol drinking. ^d^ Model 3: additionally, adjusted for opium use, history of diabetes, and history of hypertension.

**Table 3:** Hazard ratios for cause-specific-mortality, according to the dAGE quintiles ^a^.

Causes of Death	dAGE Value					
						*p*-Value
	Q1	Q2	Q3	Q4	Q5	For Trend
Cardiovascular disease						
Women						
No. of deaths	473	378	307	276	338	
Model 1	1.00	0.96 (0.84, 1.10)	0.77 (0.66, 0.89)	0.70 (0.60, 0.81)	0.77 (0.68, 0.88)	<0.001
Model 2	1.00	0.97 (0.85, 1.11)	0.78 (0.68, 0.90)	0.72 (0.62, 0.84)	0.83 (0.71, 0.96)	<0.001
Model 3	1.00	1.01 (0.88, 1.16)	0.82 (0.71, 0.94)	0.74 (0.64, 0.87)	0.81 (0.70, 0.94)	<0.001
Men						
No. of deaths	448	415	382	348	397	
Model 1	1.00	0.96 (0.84, 1.10)	0.90 (0.79, 1.04)	0.80 (0.70, 0.92)	0.92 (0.81, 1.06)	0.030
Model 2	1.00	0.98 (0.86, 1.13)	0.91 (0.79, 1.05)	0.82 (0.71, 0.95)	0.95 (0.82, 1.09)	0.064
Model 3	1.00	1.01 (0.88, 1.15)	0.93 (0.81, 1.07)	0.83 (0.72, 0.96)	0.94 (0.81, 1.08)	0.066
All						
Model 1	1.00	0.97 (0.88, 1.07)	0.84 (0.76, 0.93)	0.76 (0.69, 0.84)	0.85 (0.77, 0.94)	<0.001
Model 2	1.00	0.98 (0.89, 1.08)	0.85 (0.77, 0.94)	0.78 (0.70, 0.86)	0.89 (0.81, 0.99)	<0.001
Model 3	1.00	1.01 (0.92, 1.11)	0.88 (0.80, 0.97)	0.79 (0.71, 0.88)	0.88 (0.79, 0.98)	<0.001
All cancer						
Women						
No. of deaths	165	140	156	145	149	
Model 1	1.00	0.97 (0.77, 1.21)	1.06 (0.85, 1.32)	1.00 (0.80, 1.25)	0.96 (0.77, 1.19)	0.914
Model 2	1.00	0.95 (0.76, 1.19)	1.03 (0.82, 1.29)	0.97 (0.77, 1.22)	0.90 (0.71, 1.14)	0.832
Model 3	1.00	0.96 (0.76, 1.20)	1.04 (0.83, 1.29)	0.97 (0.77, 1.22)	0.90 (0.71, 1.14)	0.811
Men						
No. of deaths	222	208	199	203	194	
Model 1	1.00	0.95 (0.79, 1.15)	0.93 (0.76, 1.12)	0.92 (0.76, 1.11)	0.89 (0.73, 1.08)	0.788
Model 2	1.00	0.96 (0.79, 1.16)	0.93 (0.77, 1.13)	0.92 (0.76, 1.12)	0.88 (0.73, 1.09)	0.820
Model 3	1.00	0.96 (0.80, 1.17)	0.94 (0.77, 1.14)	0.92 (0.76, 1.12)	0.87 (0.71, 1.07)	0.747
All						
Model 1	1.00	0.98 (0.85, 1.14)	1.00 (0.87, 1.15)	0.98 (0.84, 1.13)	0.92 (0.80, 1.07)	0.835
Model 2	1.00	0.96 (0.83, 1.11)	0.98 (0.84, 1.13)	0.94 (0.81, 1.09)	0.90 (0.77, 1.04)	0.681
Model 3	1.00	0.96 (0.83, 1.12)	0.98 (0.85, 1.14)	0.95 (0.82, 1.10)	0.89 (0.76, 1.03)	0.594
Gastrointestinal cancer						
Women						
No. of deaths	75	65	77	78	67	
Model 1	1.00	1.02 (0.73, 1.43)	1.19 (0.87, 1.64)	1.23 (0.89, 1.68)	0.95 (0.69, 1.33)	0.454
Model 2	1.00	1.01 (0.72, 1.41)	1.16 (0.84, 1.61)	1.19 (0.86, 1.65)	0.91 (0.64, 1.29)	0.449
Model 3	1.00	1.02 (0.73, 1.42)	1.17 (0.85, 1.62)	1.20 (0.87, 1.66)	0.91 (0.64, 1.28)	0.410
Men						
No. of deaths	127	119	120	128	110	
Model 1	1.00	0.96 (0.75, 1.23)	0.98 (0.76, 1.26)	1.02 (0.80, 1.30)	0.88 (0.68, 1.14)	0.843
Model 2	1.00	0.95 (0.74, 1.23)	0.98 (0.76, 1.26)	1.00 (0.78, 1.29)	0.87 (0.67, 1.14)	0.813
Model 3	1.00	0.96 (0.74, 1.23)	0.99 (0.77, 1.27)	1.00 (0.79, 1.30)	0.86 (0.66, 1.12)	0.748
All						
Model 1	1.00	1.01 (0.83, 1.24)	1.08 (0.89, 1.32)	1.13 (0.93, 1.37)	0.92 (0.76, 1.13)	0.348
Model 2	1.00	0.98 (0.80, 1.19)	1.05 (0.86, 1.29)	1.08 (0.88, 1.31)	0.89 (0.72, 1.10)	0.409
Model 3	1.00	0.98 (0.80, 1.20)	1.06 (0.87, 1.29)	1.08 (0.89, 1.32)	0.89 (0.72, 1.09)	0.339
Other cancers						
Women						
No. of deaths	90	75	79	67	82	
Model 1	1.00	0.92 (0.68, 1.25)	0.95 (0.70, 1.29)	0.82 (0.60, 1.13)	0.96 (0.71, 1.29)	0.813
Model 2	1.00	0.90 (0.66, 1.23)	0.92 (0.68, 1.25)	0.79 (0.57, 1.09)	0.89 (0.65, 1.23)	0.714
Model 3	1.00	0.90 (0.66, 1.23)	0.92 (0.68, 1.26)	0.79 (0.57, 1.09)	0.89 (0.65, 1.23)	0.720
Men						
No. of deaths	95	89	79	75	84	
Model 1	1.00	0.95 (0.71, 1.27)	0.85 (0.63, 1.15)	0.79 (0.58, 1.07)	0.89 (0.66, 1.19)	0.576
Model 2	1.00	0.97 (0.72, 1.30)	0.87 (0.64, 1.18)	0.80 (0.59, 1.09)	0.91 (0.67, 1.24)	0.649
Model 3	1.00	0.98 (0.73, 1.31)	0.87 (0.65, 1.18)	0.80 (0.59, 1.09)	0.90 (0.66, 1.22)	0.651
All						
Model 1	1.00	0.95 (0.77, 1.17)	0.91 (0.74, 1.13)	0.82 (0.66, 1.02)	0.92 (0.75, 1.14)	0.478
Model 2	1.00	0.94 (0.76, 1.16)	0.89 (0.72, 1.11)	0.80 (0.64, 1.00)	0.90 (0.72, 1.12)	0.377
Model 3	1.00	0.94 (0.76, 1.17)	0.90 (0.73, 1.12)	0.80 (0.64, 1.00)	0.89 (0.71, 1.11)	0.387
Respiratory disease						
Women						
No. of deaths	72	47	52	43	56	
Model 1	1.00	0.79 (0.54, 1.13)	0.85 (0.59, 1.22)	0.71 (0.49, 1.03)	0.83 (0.58, 1.17)	0.453
Model 2	1.00	0.85 (0.59, 1.23)	0.96 (0.67, 1.38)	0.85 (0.57, 1.26)	1.09 (0.74, 1.59)	0.680
Model 3	1.00	0.86 (0.60, 1.25)	0.97 (0.67, 1.40)	0.85 (0.58, 1.26)	1.09 (0.75, 1.58)	0.719
Men						
No. of deaths	107	90	45	77	59	
Model 1	1.00	0.88 (0.67, 1.17)	0.45 (0.32, 0.64)	0.75 (0.56, 1.00)	0.58 (0.42, 0.80)	<0.001
Model 2	1.00	0.99 (0.75, 1.31)	0.53 (0.37, 0.75)	0.91 (0.67, 1.23)	0.76 (0.55, 1.07)	0.004
Model 3	1.00	0.99 (0.75, 1.32)	0.53 (0.37, 0.75)	0.90 (0.66, 1.21)	0.74 (0.53, 1.03)	0.003
All						
Model 1	1.00	0.87 (0.70, 1.09)	0.62 (0.48, 0.79)	0.76 (0.60, 0.95)	0.68 (0.54, 0.86)	0.001
Model 2	1.00	0.94 (0.75, 1.18)	0.70 (0.54, 0.89)	0.89 (0.70, 1.13)	0.89 (0.69, 1.14)	0.074
Model 3	1.00	0.95 (0.76, 1.19)	0.70 (0.54, 0.90)	0.88 (0.70, 1.12)	0.86 (0.67, 1.10)	0.073
Infectious disease						
Women						
No. of deaths	57	35	36	33	38	
Model 1	1.00	0.76 (0.50, 1.15)	0.76 (0.50, 1.16)	0.70 (0.46, 1.08)	0.71 (0.47, 1.08)	0.395
Model 2	1.00	0.79 (0.52, 1.21)	0.82 (0.53, 1.25)	0.77 (0.49, 1.20)	0.83 (0.54, 1.29)	0.748
Model 3	1.00	0.80 (0.53, 1.23)	0.83 (0.54, 1.26)	0.78 (0.50, 1.21)	0.82 (0.53, 1.28)	0.779
Men						
No. of deaths	53	38	40	48	40	
Model 1	1.00	0.75 (0.50, 1.14)	0.81 (0.54, 1.22)	0.94 (0.64, 1.40)	0.80 (0.53, 1.20)	0.609
Model 2	1.00	0.79 (0.52, 1.20)	0.87 (0.57, 1.31)	1.02 (0.68, 1.53)	0.90 (0.58, 1.38)	0.740
Model 3	1.00	0.80 (0.52, 1.21)	0.88 (0.58, 1.33)	1.03 (0.69, 1.53)	0.87 (0.57, 1.34)	0.753
All						
Model 1	1.00	0.76 (0.56, 1.02)	0.79 (0.59, 1.06)	0.84 (0.63, 1.11)	0.76 (0.57, 1.01)	0.268
Model 2	1.00	0.78 (0.58, 1.06)	0.83 (0.62, 1.12)	0.90 (0.67, 1.21)	0.86 (0.63, 1.17)	0.556
Model 3	1.00	0.80 (0.59, 1.07)	0.84 (0.63, 1.13)	0.91 (0.68, 1.22)	0.84 (0.62, 1.14)	0.592
Injuries						
Women						
No. of deaths	27	25	27	21	30	
Model 1	1.00	0.99 (0.57, 1.71)	1.05 (0.62, 1.79)	0.84 (0.47, 1.48)	1.16 (0.69, 1.96)	0.846
Model 2	1.00	1.03 (0.59, 1.78)	1.11 (0.64, 1.91)	0.90 (0.50, 1.63)	1.28 (0.73, 2.24)	0.796
Model 3	1.00	1.04 (0.60, 1.79)	1.12 (0.65, 1.92)	0.92 (0.51, 1.65)	1.28 (0.73, 2.24)	0.814
Men						
No. of deaths	58	50	48	59	57	
Model 1	1.00	0.80 (0.55, 1.17)	0.77 (0.53, 1.13)	0.92 (0.64, 1.32)	0.90 (0.62, 1.29)	0.669
Model 2	1.00	0.81 (0.55, 1.18)	0.78 (0.53, 1.15)	0.93 (0.64, 1.34)	0.90 (0.62, 1.33)	0.694
Model 3	1.00	0.81 (0.55, 1.19)	0.79 (0.53, 1.16)	0.93 (0.64, 1.35)	0.90 (0.61, 1.32)	0.722
All						
Model 1	1.00	0.89 (0.65, 1.21)	0.88 (0.65, 1.20)	0.94 (0.69, 1.28)	1.02 (0.75, 1.37)	0.850
Model 2	1.00	0.87 (0.64, 1.19)	0.87 (0.64, 1.20)	0.92 (0.67, 1.26)	1.01 (0.74, 1.38)	0.799
Model 3	1.00	0.88 (0.64, 1.20)	0.88 (0.64, 1.20)	0.93 (0.68, 1.27)	1.00 (0.73, 1.37)	0.844
Other causes						
Women						
No. of deaths	178	155	133	129	139	
Model 1	1.00	1.06 (0.85, 1.32)	0.89 (0.71, 1.12)	0.87 (0.69, 1.09)	0.84 (0.67, 1.04)	0.202
Model 2	1.00	1.05 (0.84, 1.30)	0.88 (0.70, 1.10)	0.85 (0.68, 1.08)	0.83 (0.65, 1.05)	0.202
Model 3	1.00	1.09 (0.87, 1.35)	0.90 (0.72, 1.14)	0.88 (0.69, 1.10)	0.81 (0.64, 1.03)	0.137
Men						
No. of deaths	191	167	139	147	149	
Model 1	1.00	0.90 (0.73, 1.11)	0.76 (0.61, 0.95)	0.78 (0.63, 0.97)	0.80 (0.65, 1.00)	0.071
Model 2	1.00	0.89 (0.72, 1.10)	0.75 (0.60, 0.93)	0.76 (0.61, 0.95)	0.77 (0.61, 0.96)	0.039
Model 3	1.00	0.91 (0.74, 1.13)	0.77 (0.61, 0.95)	0.78 (0.63, 0.97)	0.76 (0.60, 0.94)	0.041
All						
Model 1	1.00	0.98 (0.85, 1.14)	0.83 (0.71, 0.97)	0.84 (0.72, 0.98)	0.83 (0.71, 0.97)	0.018
Model 2	1.00	0.97 (0.83, 1.12)	0.81 (0.69, 0.95)	0.81 (0.69, 0.95)	0.80 (0.68, 0.94)	0.008
Model 3	1.00	0.99 (0.86, 1.16)	0.83 (0.71, 0.98)	0.83 (0.71, 0.97)	0.79 (0.67, 0.92)	0.005

DAGE, dietary advanced glycated end product; Q, quintile. ^a^ Cox proportional hazards regression models for estimating HRs and 95% CIs. Model 1: adjusted for age. Model 2: additionally, adjusted for gender (except when stratified by gender), energy intake, physical activity, smoking, BMI, and alcohol drinking. Model 3: additionally, adjusted for opium use, history of diabetes, and history of hypertension.

## Data Availability

The data presented in this study are available on request from the corresponding author.
